# The Origins of Fashion

**DOI:** 10.1002/evan.70039

**Published:** 2026-07-01

**Authors:** Francesco d'Errico, Solange Rigaud

**Affiliations:** ^1^ CNRS, Univ. Bordeaux, UMR 5199 PACEA, Batiment B2 Créteil France; ^2^ Department of Archaeology, History, Cultural Studies and Religion, Centre for Early Sapiens Behaviour (SapienCE) University of Bergen Bergen Norway

**Keywords:** body modification, cultural evolution, distributed cognition, epistemic niche construction, identity signaling, personal ornaments, social information

## Abstract

This paper reconceptualizes fashion as a deep‐time system of bodily communication rather than a byproduct of modern consumer societies. We define fashion as a socially transmitted system of bodily display in which patterned variation occurs within shared conventions of appearance. Archaeologically, fashion becomes identifiable when recurring forms of body modification and ornamentation coexist with socially meaningful differences in their selection, combination, and arrangement. From this perspective, fashion emerged through the long‐term culturalization of the human body, whereby appearance became an increasingly important medium for communicating identity, affiliation, status, and social relationships. Archaeological evidence suggests that this process began at least 400,000 years ago with pigment use, intensified with the emergence of personal ornaments, and developed into regionally differentiated systems of bodily display during the Upper Paleolithic, Neolithic, and Metal Ages. Drawing on archaeological, anthropological, and cognitive approaches, we interpret this trajectory as a cultural evolutionary process involving the generation of novel forms of bodily variation, their transmission through socially learned conventions, and their persistence, transformation, or disappearance under changing social and economic conditions. We argue that fashion functioned as an externalized social memory system that reduced cognitive demands by embedding social information in material form. In this sense, fashion emerged as a cultural technology that made social information increasingly visible, interpretable, and transmissible through bodily display.

## 1 Introduction

Fashion is often treated as a distinctly modern phenomenon, ephemeral, commercial, and driven by cycles of novelty [1, 2, 3]. In this view, fashion is typically associated with industrial capitalism, consumer culture, and rapidly changing styles. Yet such definitions become problematic when one seeks to investigate fashion‐like phenomena in deep time, where the materials under consideration are not textiles or garments but pigments, beads, shells, and bodily modifications. Before tracing the evolutionary history of fashion, a working definition is therefore required, one that is theoretically grounded, operational, and applicable to archaeological evidence.

Over the last century, fashion theory has produced a wide range of definitions, each emphasizing different dimensions of the phenomenon. Classic sociological approaches define fashion as emerging from the tension between imitation and differentiation [3] or as the outcome of a continuous process of collective taste formation [1]. Semiotic perspectives conceptualize fashion as a language or structured code through which garments communicate meaning [4], while institutional approaches emphasize fashion as the system that produces, legitimizes, and evaluates clothing rather than clothing itself [2]. More recent embodied perspectives stress that fashion is something people do with their bodies, producing lived, situated identities through material practice [5].

Although these definitions differ in emphasis, they converge on several key features. Across theoretical traditions, fashion is consistently understood as involving shared conventions that render appearance intelligible within a group; variation and innovation that allow differentiation at individual or subgroup levels; social meaning related to identity, status, or aspiration; embodiment, insofar as fashion operates on and through the body; and collective dynamics, since fashion is learned, transmitted, negotiated, and evaluated socially. These perspectives reveal fashion not as a superficial aesthetic practice but as a complex social system that regulates how bodies become meaningful in interaction.

At the same time, archaeological and anthropological research has increasingly recognized that permanent and non‐permanent practices devoted to the culturalization of the human body, such as dress, personal ornamentation, body painting, hairstyles, tattooing, scarification, and bodily implants, constitute fundamental human communication technologies. A substantial body of work has demonstrated that ornaments, dress, and other forms of bodily display can convey information about social identity, affiliation, status, reciprocity, social relationships, and group boundaries, with implications for cognition, demography, and social organization. Archaeological approaches to style have been particularly influential in this regard. Wobst [6] proposed that stylistic variation functions as a medium of information exchange among social groups. Building on these perspectives, Wiessner [7] distinguished between emblemic styles that communicate group membership and assertive styles that signal individual identity. Hodder [8] further argued that material culture participates in the negotiation and reproduction of social relationships and boundaries. Such communication need not be restricted to identity signaling and may also concern affiliation, alliance formation, reciprocity, participation in exchange networks, or other forms of social relationships.

At the same time, not all variation in bodily adornment necessarily reflects intentional communication. Dunnell [9] famously argued that style and function constitute distinct dimensions of material culture and proposed that stylistic variation may arise and persist without serving a communicative role. Likewise, Bourdieu's [10] concept of habitus highlights how socially learned practices can generate patterned differences without conscious strategic intent, while Sackett's [11, 12] notion of isochrestic variation emphasizes that some stylistic differences arise from culturally transmitted traditions rather than deliberate signaling. These perspectives caution against assuming that every observable difference in ornaments, dress, or bodily modification was intended to communicate identity, status, or affiliation. The distinction is important because archaeological research has generally treated bodily adornment, style, and symbolic communication as part of a broad category of material signaling behavior. Much less attention has been devoted to identifying the specific conditions under which bodily display becomes fashion. As a result, concepts such as style, ornamentation, identity signaling, and fashion are often used interchangeably, despite referring to different phenomena.

In this paper, we define fashion as a socially transmitted system of bodily display that communicates social information while allowing innovation and socially recognized variation within shared conventions. Two properties are central to this definition. First, fashion relies on bodily modification or adornment as a medium of communication. Second, fashion tolerates and interprets variation within a recognizable symbolic framework. It is this second feature that distinguishes fashion from many other forms of body culturalization, including rigid status insignia, fixed scarification traditions, or stylistic conventions maintained primarily through habitus or isochrestic transmission. While all fashion is style, not all style is fashion. Fashion emerges when departures from established norms become socially meaningful without undermining the recognizability of the system itself. By “negotiated” we refer to variation that is socially tolerated, interpreted, and reproduced within a community rather than being entirely prescribed by rigid rules or left to unrestricted individual choice. In this sense, negotiation does not imply explicit deliberation but the continual adjustment of appearance within socially recognized boundaries. Fashion concerns variation within a shared system of appearance; status signaling concerns the communication of social rank. The two often overlap but are not equivalent.

Archaeologically, these properties must be inferred from recurring patterns in bodily display. Shared conventions are indicated by the repeated use of the same ornament types, body locations, or display practices within a population or cultural tradition. Variation is inferred when individuals differ in the quantity, combination, arrangement, or association of these elements while still participating in the same broader system of appearance. In other words, throughout the paper, we evaluate fashion archaeologically through three criteria: (1) evidence for shared display conventions, (2) evidence for patterned inter‐individual variation, and (3) indications that such variation was socially tolerated rather than exclusively determined by status, age, sex, or ritual prescription. Fashion becomes archaeologically identifiable when patterned variation occurs within a recognizable framework of shared practices, rather than through complete uniformity or unrestricted idiosyncrasy.

This distinction is particularly important in archaeological contexts. Evidence for ornaments, pigments, clothing, or bodily modification does not automatically imply the existence of fashion. Rather, fashion must be inferred when the forms of variation described above recur within recognizable systems of bodily display and cannot be explained solely by functional constraints, inherited traditions, or rigid prescriptions. Our objective is therefore not to replace existing theories of style or symbolic communication, but to identify the conditions under which bodily style becomes fashion sensu stricto and to explore when those conditions first emerged in human evolution. From a cultural evolutionary perspective, the sequence explored here involves the emergence of novel forms of variation in bodily display, their transmission through socially learned conventions, and their differential persistence, modification, or disappearance under changing demographic, social, and economic conditions. Understood in this way, fashion does not emerge as an isolated cultural invention. Rather, it develops within a much broader evolutionary process that we refer to as the culturalization of the human body: the long‐term transformation of the body from a primarily biological substrate into a socially encoded medium. Drawing on archaeological evidence from pigments, clothing technologies, personal ornaments, and later textile‐based display systems, we argue that this process laid the foundation for fashion as a communicative system. Available evidence suggests that the culturalization of the body began at least 400,000 years ago (ka), when mineral pigments were first systematically collected and modified for both functional and symbolic purposes [13], and intensified with the emergence of personal ornaments around 160 ka in Africa [14, 15, 16, 17].

Our approach builds on niche‐construction and epistemic niche‐construction theories, which emphasize that organisms actively modify both their environments and the selection pressures acting on them. In humans, these modifications increasingly take informational and symbolic forms, creating cultural environments that influence learning, social interaction, and the transmission of behavior across generations [18]. From this perspective, the body becomes a primary surface for the externalization of cognitive tools. Once decorated, modified, or extended through pigments, beads, clothing, and permanent alterations, the body functions as a mobile interface for social information: a visible, durable, and interpretable medium through which identity, affiliation, and difference are communicated.

From an evolutionary perspective, fashion presents a paradox. Human societies rely on cumulative culture, which depends on the faithful transmission of shared, evolving conventions. If bodily displays function as communicative signals, stability and recognizability would appear advantageous. Yet fashion introduces variation, innovation, and even ambiguity into these codes, seemingly undermining their communicative reliability. This paradox becomes intelligible when considered in light of the emergence of what Moffett [19] calls anonymous societies, large‐scale social systems in which individuals must recognize one another as members of the same community without relying on personal familiarity or kinship ties. In such social contexts, rigid uniformity of appearance would limit the capacity to signal roles, reputations, or aspirations, while excessive variation would obscure group membership. Fashion resolves this tension by establishing shared conventions that ensure recognizability while simultaneously allowing bounded variation through which individuals negotiate identity, express status, or signal affiliations. In this sense, fashion is not a disruption of symbolic communication but a regulatory mechanism that sustains cohesion in large‐scale societies by balancing conformity with controlled differentiation.

**Figure 1:**
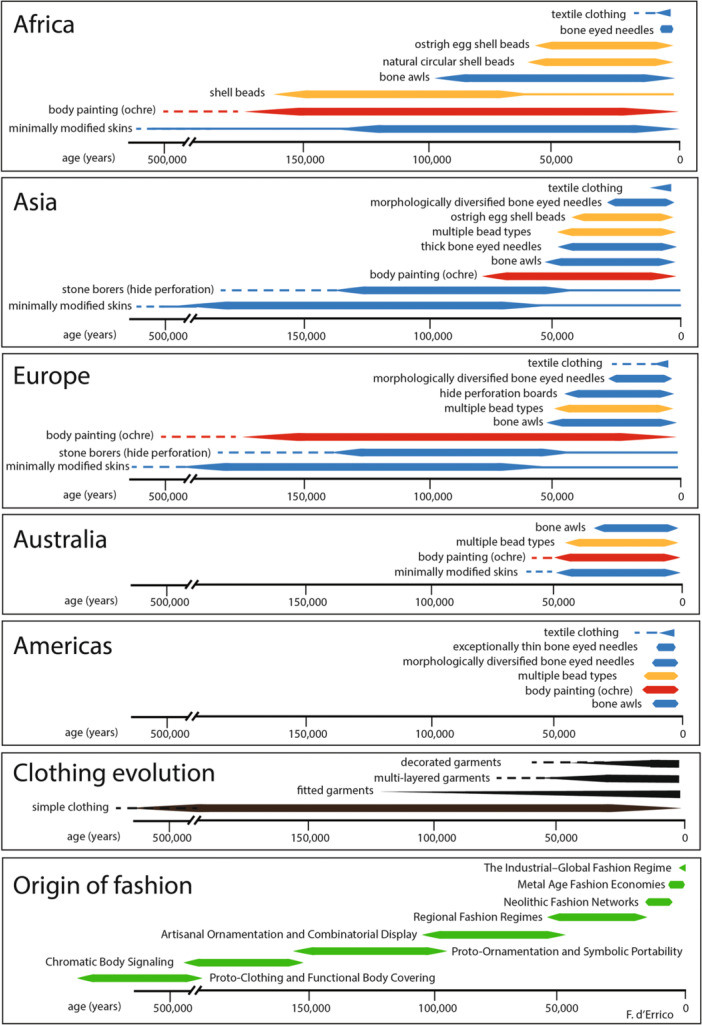
This figure integrates archaeological proxies for clothing technologies (blue), pigment‐based body modification (red), and personal ornamentation (orange) across major world regions. Temporal ranges are schematic; dashed segments indicate inferred or poorly documented phases, and tapered bar ends reflect gradual intensification or decline. Early hide‐working and body‐covering mark the onset of body culturalization, initially driven by functional protection. Systematic ocher use documents the emergence of chromatic body signaling, transforming appearance into a social medium. Proto‐ornamentation begins with the suspension of natural objects and shell beads, introducing symbolic portability. The manufacture and diversification of fully worked beads signal artisanal ornamentation and the rise of combinatorial display systems. Sewing and tailoring technologies, traced through the emergence and diversification of bone‐eyed needles and hide‐perforation tools, enable fitted and composite garments, expanding the body as a surface for social differentiation. The figure highlights that fashion emerges from the long‐term convergence of clothing, ornamentation, and pigment use into socially regulated systems of bodily display in which variation among individuals becomes increasingly visible and socially meaningful within shared conventions.

In what follows, we bring together archaeological, anthropological, and neurocognitive evidence, drawn primarily from African and European records, to outline an evolutionary framework for fashion as a material form of communication. We trace this development across several key thresholds: the emergence of protective clothing technologies as early as 700 ka; ocher use and its intensification from around 400 ka in Africa and Europe; the appearance and diversification of personal ornaments from approximately 160 ka; the proliferation of composite body displays during the Upper Paleolithic; the standardization and expansion of ornament traditions in the Neolithic; and the growing entanglement of bodily appearance with systems of social inequality, exchange, and market economies from the Metal Ages to the present. Across this *longue durée*, we show that fashion emerges not as a sudden invention but as a threshold phenomenon: once the body becomes a site of iterative distinction within shared symbolic codes, social life is transformed. Individuals acquire portable identities, social differences become increasingly visible, and coordination extends beyond kinship through appearance alone. In this sense, fashion became an increasingly important component of human social evolution. The evolutionary sequence outlined below is summarized schematically in Figure 1. Rather than a linear progression toward modern fashion, this sequence documents successive thresholds in the culturalization of the human body, each expanding the communicative capacity of appearance. The following sections correspond to these phases and describe how clothing, pigments, and ornaments gradually converged into socially regulated fashion systems.

## 2 Proto‐Clothing and the Functional Culturalization of the Body

To understand the emergence of fashion as a communicative system, it is necessary to begin with the earliest phase in the culturalization of the body (Figure 1), when bodily coverings or modifications served primarily functional purposes but created the material conditions for later symbolic display. The earliest clothing, likely made from hides, furs, or plant fibers, was almost certainly adopted for protection against environmental stress [14, 20, 21]. However, even purely functional systems can acquire secondary signaling value once they become regularly visible markers of identity, especially when variation emerges across groups or individuals. This is the foundation of body culturalization: when material solutions to practical needs become embedded in social logics, reinterpreted as indicators of affiliation, role, or status.

Clothing made of leather or plant fibers rarely survives beyond a few thousand years, making the earliest sartorial practices archaeologically elusive. Some form of body covering may already have been required during the first hominin dispersals out of Africa around 2 Ma [22, 23], which entailed a substantial expansion into cooler and more seasonal environments at lower and middle latitudes of Eurasia. No direct or indirect evidence allows us to characterize the nature, extent, or cultural significance of such early clothing. However, several independent lines of indirect evidence point to the existence of body‐covering technologies far earlier than their material traces allow us to see directly. Use‐wear on stone tools, diagnostic cutmarks on animal bones indicating the careful removal of whole hides, specialized bone tools for skin processing [24, 25, 26], and genetic divergence between body and head lice [27, 28], all suggest that proto‐clothing for protection against cold and windchill was already in place by 700 ka, and likely earlier. Once clothing became a habitual and socially visible component of the body, it created new surfaces for variation, comparison, and social interpretation. Even when initially adopted for protection, repeated exposure to choice points, such as material selection, cut, fastening, or repair, would have allowed clothing to acquire secondary social meanings, thereby extending body culturalization beyond ornamentation to the dressed body itself.

**Figure 2:**
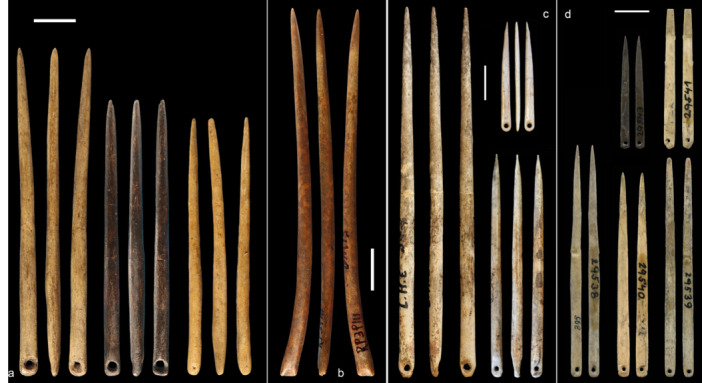
The earliest know bone eyed needles, found at Xiaogushan (a) and Zhoukoudian Upper Cave (b), China, compared with Magdalenian needles from France (c) and Moravia (d). Scale = 1 cm. *Source:* Adapted from d'Errico et al. [29].

In southern Africa, slender bone awls for piercing hides and, possibly, for tattooing, appear around 90 ka [29], while in Europe, techniques for puncturing leather using bone punching boards are documented from 36 ka [30]. The earliest bone‐eyed needles (Figure 2), equipped with drilled threading apertures, emerge in East Asia around 40 ka, only reaching Europe circa 26 ka [21, 29].

Following their initial adoption, eyed needles underwent two parallel evolutionary trajectories over the next 30,000 years: a progressive refinement resulting in increasingly finer forms, and a diversification into a wide range of sizes, indicating a broadening of functions. This dual trend suggests that eyed needles were no longer used solely for assembling outer garments, but also for attaching ornaments to clothing, sewing fitted or delicate garments such as underwear, and integrating composite body displays, marking a decisive expansion in the culturalization of dress [14, 21].

## 3 Chromatic Body Signaling

The earliest material evidence that may signal body culturalization comes from ocher, iron‐rich mineral pigments that were collected, ground, and transported by hominins as early as 400,000 years ago at sites such as Maastricht‐Belvédère and Terra Amata in Europe, and Kathu Pan 1, Wonderwerk Cave, and Kapthurin in Africa [31, 32, 33, 34, 35]. This stage marks the first transformation of the body into an intentional visual medium. While some early uses of ocher were possibly functional, serving as insect repellent, sunscreen, hide preservative, or adhesive component [36, 37], quantitative data from a recent large‐scale database of African Middle Stone Age sites show that ocher use increases around 320 ka, followed by a second, more striking tipping point at 160 ka [13]. The latter has been interpreted as evidence that from this stage onward, ocher was consistently employed for signaling or symbolic purposes. Further support for a communicative role comes from technological behaviors that exceed functional necessity. At several African and Levantine sites, yellow goethite‐rich ocher was deliberately heated to produce red hematite pigments, indicating controlled chromatic preference, while high‐quality ocher was transported over long distances, suggesting selective sourcing for aesthetic or social reasons. Crucially, systematic application of ocher to modify the color of personal ornaments is already documented by 140,000 years ago [38], demonstrating that pigments were not also used on bodies on accessories intended to be displayed, implying deliberate chromatic signaling [39]. Recent evidence from Neanderthal contexts in Crimea reinforces this trend of intentional chromatic signaling beyond *Homo sapiens*. At sites such as Zaskalnaya VI, Neanderthals shaped and repeatedly resharpened ocher crayons, whose wear patterns indicate they were used to draw red lines or abstract designs on soft materials, possibly human skin or clothing. Such practices imply shared visual code recognizable within their communities [40].

Even if these early pigment practices do not yet constitute full fashion systems, the deliberate placement of color on bodies or garments represents a crucial shift: appearance becomes a social medium, setting the stage for later expansions of body culturalization.

## 4 Proto‐Ornamentation and Symbolic Portability

**Figure 3:**
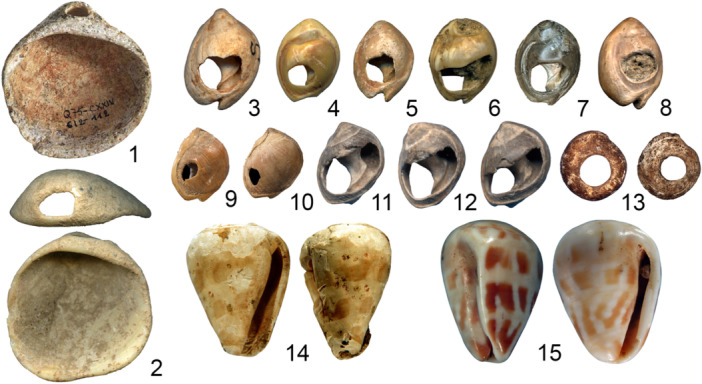
Early personal ornaments: (1) *Glycimeris* shell with traces of red pigment and a natural perforation, Qafzeh, Israel, c. 95 ka; (2) *Glycimeris insubrica* shell with a natural perforation, Cueva de Los Aviones, Spain, c. 115 ka; (3) *Tritia gibbosula* shell with a natural perforation, Skhul, Israel, c. 115 ka 2; (4–5) Grotte de Pigeons, Taforalt, Morocco, c. 80 ka; (6) *T. gibbosula* shell with a natural perforation, Rhafas, Morocco, c. 80 ka; (7) *T. gibbosula* shell with a natural perforation, Ifri N'Ammar, Morocco, c. 120 ka. Note the dark coloring of the shell, most likely due to deliberate heating; (8) *T. gibbosula* shell with a natural perforation, Oued Djebbana, Algeria; (9–12) *Nassarius kraussianus* shells, Blombos Cave, South Africa, c. 73 ka. Note the difference in perforation size and type between shell beads 9–10 and 11–12. The dark coloring of the latter is due to heating; (13) naturally perforated Conus apexes used as beads, Panga ya Saidi, Kenya, c. 60–40 ka; (14) *Conus ebraeus* shell bead associated with the burial of an infant, Border Cave, KwaZulu‐Natal, South Africa, c. 74 ka; (15) *C. ebraeus* shell bead, Border Cave, KwaZulu‐Natal, South Africa, c. 74 ka.

The emergence of personal ornaments was not a singular invention but a long evolutionary process unfolding over tens of millennia, marked by increasingly deliberate manipulations of material for signaling effect (Figure 1). The earliest evidence, beginning around 160 ka in North Africa, 110 ka in the Levant, 90 ka in southern Africa, and 130 ka in Europe, consists of naturally perforated marine shells, typically belonging to the same or closely related species, which were selected, collected, and suspended with minimal modification [14, 16, 41, 42, 43, 44, 45]. These early practices already imply shared rules of selection and visual coherence (Figure 3). By 90−70 ka, we witness a decisive shift toward intentionality: shells were now deliberately perforated, as seen at sites such as Blombos and Sibudu [46, 47], and individuals began selecting different shell species to produce contrasting visual patterns, suggesting a move from opportunistic collection to strategic aesthetic design [48].

During and toward the end of this long phase of marine shell use, additional lines of evidence point to increasingly nuanced manipulations of ornament appearance. A limited but notable diversification in the shell species selected is observed, alongside the heat treatment of shells to modify their color and their systematic coating with ocher, likewise affecting chromatic properties. These practices indicate growing attention to visual outcomes beyond shell form alone. Although the social meaning of such modifications remains difficult to establish, they suggest that some degree of individual or subgroup variation in the way shells were displayed on the body may already have occurred within otherwise highly shared ornamental traditions.

## 5 Artisanal Ornamentation and Combinatorial Display

Around 55 ka, a major technological and communicative breakthrough appears in eastern and southern Africa with the manufacture of ostrich eggshell beads (OESB) [49]. Unlike naturally perforated shells, OESB production required a labor‐intensive, multi‐stage *chaîne opératoire*, breaking the shell, trimming blanks, drilling perforations, grinding, and polishing. This represents one of the earliest fully artisanal ornament technologies, and its extraordinary temporal endurance testifies to its entrenched social value. The consistent sizing and combinatorial use of these beads in necklaces, headbands, and waistpieces reveal formalized display grammars and aesthetic standardization.

By around 31 ka, OESB assemblages begin to show clear regional differentiation in size: beads from southern Africa become significantly smaller than those from eastern Africa, indicating that shared technological traditions had begun to diverge stylistically, marking the emergence of distinct regional preferences within a common symbolic framework [49].

Meanwhile, Neanderthals, once regarded as lacking symbolic material culture, are now recognized as participants in parallel or convergent traditions of bodily display. Evidence includes raptor claws modified for suspension [50, 51, 52], naturally perforated and pigment‐stained shells [45, 53], intentional use of feathers for visual display [54, 55], and, as mentioned above, the shaping and maintenance of ocher crayons for marking skin or clothing. Toward the end of their evolutionary trajectory, some Neanderthal groups even adopted more elaborate ornamentation strategies, suggesting an escalating investment in appearance‐based communication [56]. Taken together, these practices demonstrate that body ornamentation was not an exclusive invention of *H. sapiens*, but part of a broader Late Pleistocene signaling ecology in which multiple hominin populations experimented with materialized identity [57, 58].

## 6 Regional Fashion Regimes

With the arrival and expansion of *H. sapiens* in Eurasia and Sahul, beginning at least 50 ka, Upper Paleolithic groups developed highly specialized ornament repertoires, including perforated shells, animal teeth, stone, bone, antler, amber, and mammoth ivory beads and pendants [59, 60, 61, 62, 63]. Ornament systems solidified into structured regional style regimes [60, 64]. Differences between assemblages reveal distinct local preferences, indicating that ornamentation was culturally diagnostic, encoding group‐level identities. During the Aurignacian and Gravettian periods (c. 42–23 ka), each major European region developed characteristic combinations of bead types, which were maintained with remarkable stability over thousands of years. Such enduring material associations signal not casual decorative choices but long‐term ornamental traditions, forming stable cultural geographies [65, 66, 67].

Evidence for connectivity emerges from the circulation of ornaments and raw materials beyond their zones of production, and from the selective incorporation of non‐local elements into otherwise regionally coherent repertoires. The presence of exotic items, such as Mediterranean shells in inland contexts or red deer canines transported across ecological boundaries, demonstrates episodic or sustained contacts between groups [68, 69, 70]. In this sense, Upper Paleolithic ornamentation was governed by shared regional conventions while also providing means of recognizing affiliation and difference across wider networks of interaction, allowing stylistic traditions to remain locally distinctive despite regular intergroup contacts.

Yet if these systems were so standardized, to what extent did they tolerate innovation or allow personal expression? Habitation‐site ornament assemblages, often palimpsests of lost or discarded items, cannot answer this question. Primary burials, where ornaments remain in anatomical position, offer a rare opportunity to explore this issue. However, funerary assemblages reflect decisions made by surviving community members as well as aspects of the deceased's social identity, and therefore cannot be treated as straightforward records of appearance in life. No funerary contexts can be reliably attributed to the Aurignacian (42–34 ka), but by the Gravettian (34−24 ka), numerous richly adorned burials appear across Europe. While these burials broadly conform to the regional ornament traditions seen in habitation sites, subtle yet consistent differences in the type, quantity, or placement of ornaments on individual bodies suggest room for variation within convention [65, 67, 68, 71, 72, 73, 74, 75]. These deviations, individualized arrangements within shared norms, whether reflecting choices made during life, funerary decisions, or a combination of both, may represent the earliest archaeological traces of fashion *sensu stricto*: not merely markers of belonging, but expressions of how one belonged.

These variations, differences within conformity, suggest that by the mid‐Upper Paleolithic, and probably by its very onset, ornament use was a medium for negotiating individuality, personal roles, and emerging social hierarchies. Cases such as the double and triple burials of Sungir (Russia, ~34 ka), where two subadults were buried with tens of thousands of beads, mammoth ivory spears, and lavish regalia far exceeding the grave goods of nearby adults, indicate that age or status could override demographic expectations, and that fashion could mark elevated prestige rather than simple group belonging [72, 76].

Likewise, the burial of the young adult female at Saint‐Germain‐la‐Rivière (~15.8 ka) presents one of the clearest possible examples of status‐marked fashion in deep time. The burial dates to a cold phase of the Late Glacial, when environmental conditions in southwestern France were harsh and ungulate communities were dominated by cold‐adapted taxa. Interred in a carefully constructed stone‐lined funerary structure, the woman was adorned with at least 71 perforated red deer canines, 88% of which came from young male animals, representing a minimum of 66 different individuals. Stable isotope analyses indicate that red deer did not contribute to her diet, and red deer remains are virtually absent from the site's faunal assemblage, excluding local hunting as the source of the ornaments. Finally, the exceptional nature of this burial is underscored by the fact that ornaments made from locally available materials occur in the same archaeological layers at the site but are not associated with the human interment, while contemporary burials from the region either lack ornaments altogether or contain markedly different forms of adornment. Together, these observations suggest that the canines were acquired through long‐distance exchange networks and maintained as highly valued objects of bodily display, creating a distinctive appearance that set the deceased apart from other individuals in her community. These burials demonstrate that by the Upper Paleolithic, body ornamentation operated simultaneously as a marker of collective identity and as a mechanism for expressing difference, whether of rank, reputation, or personal distinction. However, establishing whether such differences reflect fashion sensu stricto requires particular caution. Although individual differences in ornament type, quantity, and arrangement are clearly documented in Gravettian and later burials, including emblematic cases such as Sungir, most Upper Paleolithic funerary contexts consist of single primary burials, or, more rarely, double or triple interments. This demographic structure, combined with the coarse chronological resolution of the record, severely limits our ability to disentangle variation arising from enduring systems of negotiated appearance from variation driven by social rank, exceptional status, age, kinship, or singular biographical circumstances. Even burials considered broadly contemporaneous may in fact be separated by centuries, making it difficult to assess whether observed differences reflect stable fashion‐like variability operating within the same social system or diachronic change across generations.

In other words, the presence of individualized ornamentation does not, in itself, demonstrate the existence of fashion as a collectively regulated system of tolerated variation, as defined here. What these burials document with confidence is differential access to ornamented appearance; whether such differences reflect stable fashion‐like choices or socially prescribed distinctions remains difficult to assess in the absence of larger, tightly dated samples. It is precisely in this respect that Mesolithic multiple‐burial cemeteries become critical. By providing large numbers of individuals buried within the same cultural, temporal, and spatial framework, they allow variation in bodily display to be evaluated comparatively and statistically, making it possible to distinguish between rigid status coding and more flexible systems of appearance that tolerate graded and negotiable variation.

An instructive intermediate configuration between Upper Paleolithic ornament systems and later Neolithic fashion regimes is provided by the Late Mesolithic cemetery of Oleneostrovski Mogilnik in Karelia [77, 78]. Analysis of more than 170 burials reveals a highly structured distribution of body ornaments, primarily perforated animal teeth. Ornament types such as bear tusks, elk incisors, and beaver incisors are unevenly distributed among individuals and occur in patterned associations with age, sex, and the richness of funerary assemblages. At the same time, ornament sets vary in quantity and composition, producing differences between individuals without abandoning shared conventions of adornment. Oleneostrovski thus illustrates a stage in which ornament use combined strong social regulation with variation that cannot be entirely reduced to age, sex, wealth, or social position. In this respect, it provides early evidence for a fashion‐like system of bodily display.

## 7 Neolithic Fashion Networks

If the Upper Paleolithic and the Mesolithic document the emergence of localized fashion‐like dynamics, the Neolithic marks a threshold at which bodily display becomes embedded in durable, large‐scale social networks. The transition to sedentary farming after ~11 ka brought denser populations, longer‐lasting settlements, and intensified interaction, creating new conditions in which appearance could be compared, evaluated, and negotiated across communities.

**Figure 4:**
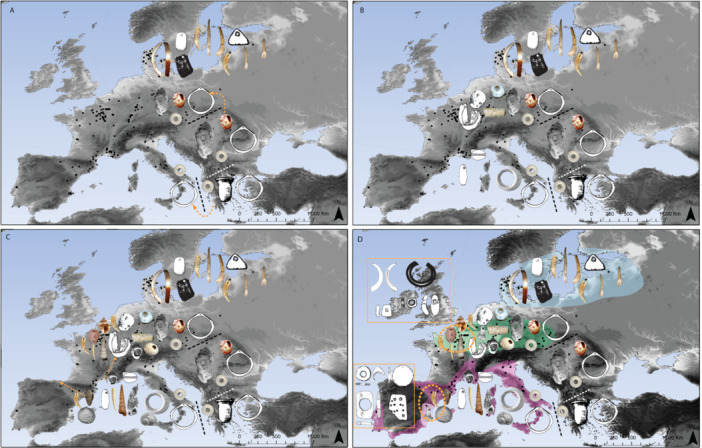
Cartography of the early Neolithic bead‐type diffusion following the dispersal of the first villagers from east to west Europe: (A) Large‐scale diffusion of exclusively Neolithic ornament types; (B) diversification of early Neolithic ornament types; (C) persistence of Mesolithic bead‐types in the Neolithic; (D) emergence of new bead types at the regional scale. Dotted black lines indicate the major shifts in bead‐type associations in South East Europe. Dotted orange ellipses show the two areas where numerous new bead‐types were adopted at the end of the early Neolithic. Color‐shaded areas indicate the geographic distribution of the Mediterranean early Neolithic (pink), Danubian early Neolithic (green), and Baltic early Neolithic (blue) archaeological cultures considered in the analysis. Maps were made by S.R. by using ArcGIS 9.3.1 software (After Rigaud et al. 2015).

In Europe, the shift from foraging to farming between 8000 and 5000 BP followed contrasting regional trajectories. Comparative analyses of Mesolithic and Early Neolithic bead assemblages show strong continuity in northern Europe, particularly around the Baltic, where bead‐type associations persist across the forager–farmer boundary [79]. This pattern suggests either resistance to cultural assimilation or the adoption of indigenous symbolic codes by incoming farmers. In contrast, Central and Southern Europe display marked turnover in ornament repertoires, with new bead forms associated with Near Eastern farming traditions introduced alongside persistent Mesolithic types (Figure 4). These changes parallel archaeological and genetic evidence for demographic movements and cannot be explained by raw‐material constraints alone.

Beyond the appearance or disappearance of bead types, Neolithic ornament systems also varied in how objects were combined and worn. Some elements of the ornament repertoire were shared across large regions, while others differed between communities and individuals, indicating that common traditions coexisted with multiple ways of assembling and displaying ornaments. This pattern is particularly clear in large cemetery data sets, such as Vedrovice (Czech Republic), where variation in grave goods, including ornaments, reflects multiple, overlapping dimensions of identity rather than a simple hierarchy [80].

Long‐distance circulation further transformed ornamentation into a medium of supra‐local communication. As ornaments circulated through exchange networks, their significance likely extended beyond identity signaling to encompass affiliation, reciprocity, and participation in wider social relationships, echoing Appadurai's (1986) view that objects acquire meaning through their circulation as much as through their possession. Cyprinid fish‐tooth ornaments appear in both Upper and Lower Danube contexts during the Late Mesolithic, sharing production techniques despite regional differences in subsistence and technology [81, 82]. Mediterranean shells such as *Columbella rustica* circulated along major river corridors, including the Rhône and Ebro, reaching inland sites where they occur in domestic and funerary contexts [82, 83]. At Große Ofnet (Germany), biometric analyses show that red deer canines originated from multiple regions, indicating symbolic economies extending well beyond local territories [84].

A defining feature of Neolithic ornament systems is the coexistence of locally produced ornaments with fully shaped objects made from non‐local raw materials. These latter items required skilled craftsmanship and long‐distance acquisition and thus carried enhanced value. Crucially, they were typically combined with locally made ornaments rather than replacing them. Variation emerged not simply from the possession of exotic items but from the ways locally produced and imported ornaments were combined and displayed.

Cemetery‐scale analyses make this dynamic particularly clear. At Vedrovice, inter‐individual variability in ornament presence, composition, and spatial distribution cannot be reduced to age, sex, or a single axis of social ranking [80]. Similar conclusions emerge from analyses of Early Neolithic cemeteries in the Paris Basin and Rhineland, where personal ornaments form structured sets whose composition and placement vary systematically between individuals within otherwise homogeneous cultural contexts [85]. Although vertical differentiation and social competition are archaeologically visible in the Linearbandkeramik [86], differences in ornament selection and arrangement often reflect additional axes of identity, such as household affiliation or life‐history trajectories.

Participation in long‐distance symbolic economies further expanded the scope for individual expression. Spondylus shells, among the earliest and most widespread Neolithic exchange goods, circulated from Mediterranean source regions to Central Europe, particularly within LBK contexts [87]. While Spondylus ornaments occur frequently in burials, they are not restricted to a narrow elite; instead, they appear in highly variable combinations with local and regional ornament types, cutting across age and sex categories [85, 88]. Their social significance thus lay less in possession than in patterned use within individual assemblages.

Evidence from the Near East further demonstrates that Neolithic systems of appearance operated at multiple levels. At Aşıklı Höyük (c. 10,350–9350 cal BP), integrated bioarchaeological analyses show that ornament assemblages followed shared conventions but varied according to age, household affiliation, and social roles rather than biological sex alone [89]. At Boncuklu Tarla, labret‐ and ear‐related ornaments associated directly with human remains provide the earliest unambiguous evidence for body piercing in the Near East, indicating that bodily modification extended beyond removable objects to include durable alterations of the body itself [90]. While these practices were culturally sanctioned and long‐lasting, variation in ornament type, material, and bodily location indicates that participation in these traditions was not completely standardized.

By the later Neolithic and early Chalcolithic, specialized ornament production reached unprecedented levels. At sites such as Kültepe I and Ovçular Tepesi, the manufacture of steatite and enstatite micro‐beads involved complex chaînes opératoires and high‐temperature firing, freeing ornament production from the constraints of natural raw‐material forms and colors [91]. These innovations expanded the expressive potential of bodily display and laid the foundations for later systems of standardized production.

In synthesis, the Neolithic represents a critical threshold in the evolution of fashion sensu stricto. Large cemeteries allow sustained comparison among contemporaries; the coexistence of local and non‐local ornaments links bodily display to exchange networks; and standardized but variably combined ornaments suggest that individuals could differentiate their appearance within widely shared conventions of adornment. The variability observed in durable ornaments likely reflects only part of a broader system of appearance that also included textiles, fibers, and color treatments that rarely preserve archaeologically. The Neolithic thus marks the consolidation of bodily display as a socially regulated yet flexible system of visual communication.

### 7.1 Metal Age Fashion Economies

In contrast to Neolithic systems of negotiated variation, the Copper Age cemetery of Varna I (c. 4550–4325 cal BC) documents bodily display in a context of pronounced social inequality. Some individuals were buried with hundreds of gold ornaments, copper objects, and exotic materials arranged on the body, whereas many others received few or none. This highly uneven distribution of prestige goods, together with evidence for specialized metallurgy and long‐distance exchange, points to marked differences in access to wealth and social status [92]. Yet the display was not organized according to a single elite template. Ornament assemblages vary considerably between richly furnished graves in the types, quantities, and combinations of objects deposited, suggesting that social distinction was expressed through multiple ways of presenting the body. Varna, therefore, provides early evidence that increasing social inequality created new opportunities for differentiating individuals within a shared symbolic system.

The Bronze Age introduces a new level of complexity to the archaeology of fashion. Ornaments are increasingly made from an alloy that is also central to the production of tools, weapons, and utilitarian objects [93, 94]. This material property profoundly affects how ornaments operate as markers of identity, wealth, and personal expression. Bronze ornaments, pins, bracelets, torcs, rings, pendants, and dress fittings became widespread across Europe, the Near East, and parts of Asia from the Early Bronze Age onward. Many of these objects are fully standardized in form, produced using moulds or repeated casting techniques, and circulate over wide regions. At the same time, their value derives not only from craftsmanship or provenance, but from their participation in broader metal economies, where copper, tin, and finished objects are acquired, exchanged, hoarded, recycled, and sometimes deliberately removed from circulation through deposition or burial [95]. This dual status of bronze, as both adornment and convertible resource, complicates the interpretation of fashion. On the one hand, metal ornaments clearly functioned as visible markers of appearance, often worn on the body or attached to garments in ways that structured silhouette, movement, and sound. On the other hand, their potential for reuse meant that wearing bronze also signaled access to material wealth that could, if necessary, be transformed into weapons, tools, or ingots. In this sense, Bronze Age fashion operates at the intersection of display and reserve: ornaments are both signs of identity and condensed stores of value [96, 97].

Archaeological contexts show that bronze ornaments were frequently combined with other materials, textiles, leather, beads of stone or amber, indicating continuity with earlier fashion logics based on assemblage and combination. Variation emerges through the selection of specific ornament types, their number, placement on the body, and their association with other materials, even when the objects themselves are morphologically standardized.

Large cemeteries and hoards provide especially clear evidence that Bronze Age fashion was socially regulated yet flexible. Within the same burial grounds, individuals differ markedly in the quantity and configuration of metal ornaments, differences that are not always reducible to age or biological sex. Some graves emphasize accumulation, others restraint; some combine multiple ornament categories, others focus on a single dominant element. These patterns suggest that appearance was negotiated within shared conventions, allowing individuals to express status, affiliation, and personal positioning through choices about what metal objects to wear, retain, or relinquish.

At the same time, the recyclability of bronze introduces a new temporal dimension to fashion. Because metal objects could be remelted, stylistic change did not necessarily require the accumulation of new raw materials, but could be driven by the reconfiguration of existing ones. This capacity likely accelerated cycles of adoption, abandonment, and recombination, while also making fashion more sensitive to economic fluctuations, access to metal sources, and control over production. Fashion in the Bronze Age thus becomes more explicitly entangled with political economy than in earlier periods.

### 7.2 The Industrial–Global Fashion Regime

The final phase of the sequence represented in Figure 1 corresponds to the Industrial–Global fashion regime, in which earlier dynamics of bodily display are amplified by mechanized production, global exchange, and mass communication. From the nineteenth century onward, textiles, garments, and ornaments were produced at unprecedented scale and circulated across planetary networks, allowing appearance to be compared, evaluated, and imitated far beyond local communities. What changes is not the cognitive structure of fashion but its temporal and spatial resolution: cycles of adoption accelerate, stylistic variation becomes more visible, and participation expands to entire populations.

From the perspective adopted here, the Industrial–Global fashion regime is best understood not as a complete rupture with earlier systems but as a large‐scale transformation of them. While modern fashion is distinguished by the rise of markets, commodification, mass production, and accelerated stylistic turnover, these developments build upon pre‐existing capacities for communicating social information through bodily display. They nevertheless transformed fashion into an increasingly autonomous sphere of consumption and social differentiation [2, 98, 99].

## 8 Cognitive Mechanisms, Epistemic Niche Construction (ENC), and the Emergence of Fashion

Understanding the emergence of fashion systems requires identifying the cognitive conditions under which bodily display could support deliberate, socially legible variation within shared conventions. At a basic level, body culturalization relies on the human capacity to rapidly extract social information from appearance. Experimental and neuroimaging studies show that faces and bodies modified with culturally salient markers, such as paint, beads, or composite adornment, engage neural networks involved in social inference, affective evaluation, and categorization, including regions associated with mentalizing, emotional salience, and symbolic processing [100]. These findings support the idea that humans are cognitively prepared to treat modified bodies as meaningful social cues rather than as neutral physical forms. Such capacities underpin the effectiveness of bodily display as a communication technology, but they do not in themselves explain fashion.

From an ENC perspective [18], the culturalization of the body can be understood as one of the earliest ways in which humans externalized social information into the environment. By adopting shared conventions of appearance, groups transformed bodies into predictable surfaces for reading identity, affiliation, or role, thereby reducing the cognitive costs of social interaction. In such systems, the body functions as a stable mnemonic and classificatory device, allowing individuals to navigate complex social worlds without relying exclusively on memory or verbal exchange. Fashion emerges when this stabilized system is pushed one step further. Fashion depends on the cognitive ability to track, compare, and evaluate differences among individuals who otherwise conform to the same visual code. This requires sensitivity to relative choices—combinations, degrees, substitutions, and departures—that remain intelligible precisely because they occur within a shared framework.

## 9 Conclusion and Future Directions

The deep‐time approach to fashion presented here suggests a profound reframing: fashion is a fundamental mode of human communication, grounded in the culturalization of the body and evolving across tens or even hundreds of millennia. What begins with ocher‐stained skin and pierced shells becomes, over time, a structured system of material signaling, capable of regulating identity, negotiating alliance, and stabilizing cooperation.

The evidence indicates that fashion becomes archaeologically identifiable when display conventions combine strong standardization with sufficiently visible variability to allow negotiated differentiation within shared symbolic frameworks. In earlier periods, such as the Middle Stone Age, the long‐term persistence of particular shell‐bead traditions indicates strong adherence to shared social conventions. However, the archaeological record provides only limited opportunities to assess the extent of contemporaneous variation among individuals. In contrast, Upper Paleolithic, Mesolithic, Neolithic, and Bronze Age ornament systems are documented by richer assemblages and funerary contexts that make inter‐individual differences more archaeologically visible. These records reveal increasingly complex, combinatorial, and situational forms of bodily display. Rather than implying the emergence of individuality itself, this trend suggests an increasing capacity to document how shared conventions accommodated variation among individuals. Upper Paleolithic and Mesolithic ornament systems represent early fashion‐like regimes, in which localized aesthetic traditions emerge. Neolithic exchange networks document the widespread circulation of ornament types that were locally combined and displayed in different ways, creating opportunities for variation within broadly shared traditions of appearance. Future work should test the framework proposed here against richer archaeological records from Asia, Oceania, and the Americas; evaluate more systematically the relationship between fashion, demographic change, and exchange networks; and develop quantitative methods for distinguishing variation attributable to social categories from variation reflecting individual differentiation within shared traditions of appearance.

## Ethics Statement

The authors confirm that this manuscript complies with the ethical standards and integrity policies of Wiley and *Evolutionary Anthropology*. The work is entirely original, has not been published previously, and is not under consideration for publication elsewhere. All sources are appropriately cited, and the authors have followed best practices regarding authorship, data interpretation, and reporting. This study does not involve experimentation on living human participants or animals. All data analyzed derive from previously published sources and/or archaeological materials curated in public or institutional collections, and their use complies with relevant legal and ethical regulations.

## Conflicts of Interest

The authors declare no conflicts of interest.

## Data Availability

Data sharing is not applicable to this article as no data sets were generated or analyzed during the current study.
